# Supplementary figures and images for: Mg, K-containing microparticle: A possible active principle of a culture extract produced by a microbial consortium
Source: PLoS One. 2021 Nov 3;16(11):e0259297. doi: 10.1371/journal.pone.0259297 (PMC8565762; doi:10.1371/journal.pone.0259297)

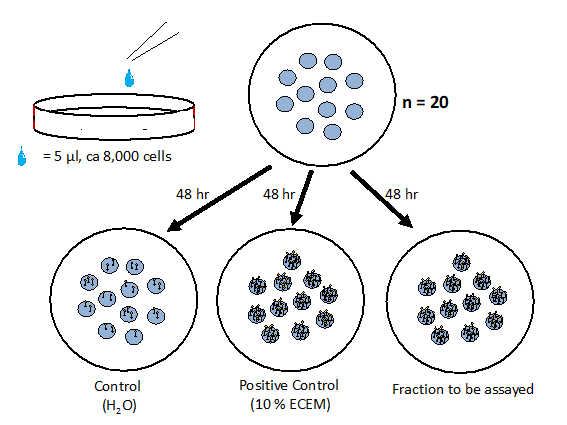

Supplement: S1 Fig — Twenty droplets of AX2 amoeba cell suspension, containing ca. 8,000 cells, were spotted onto non-nutrient agar plate which contained a separated fraction to be assayed. Forty-eight hours later, number of fruiting body was counted under dissecting microscope, and averaged to give a number for one spot. Control was composed of agar and water. Positive control contained 10% ECEM. For monitoring the active principle during purification processes, agar plate contained the separated fraction in an amount equivalent to 10% ECEM. (TIF) [file pone.0259297.s001.tif]

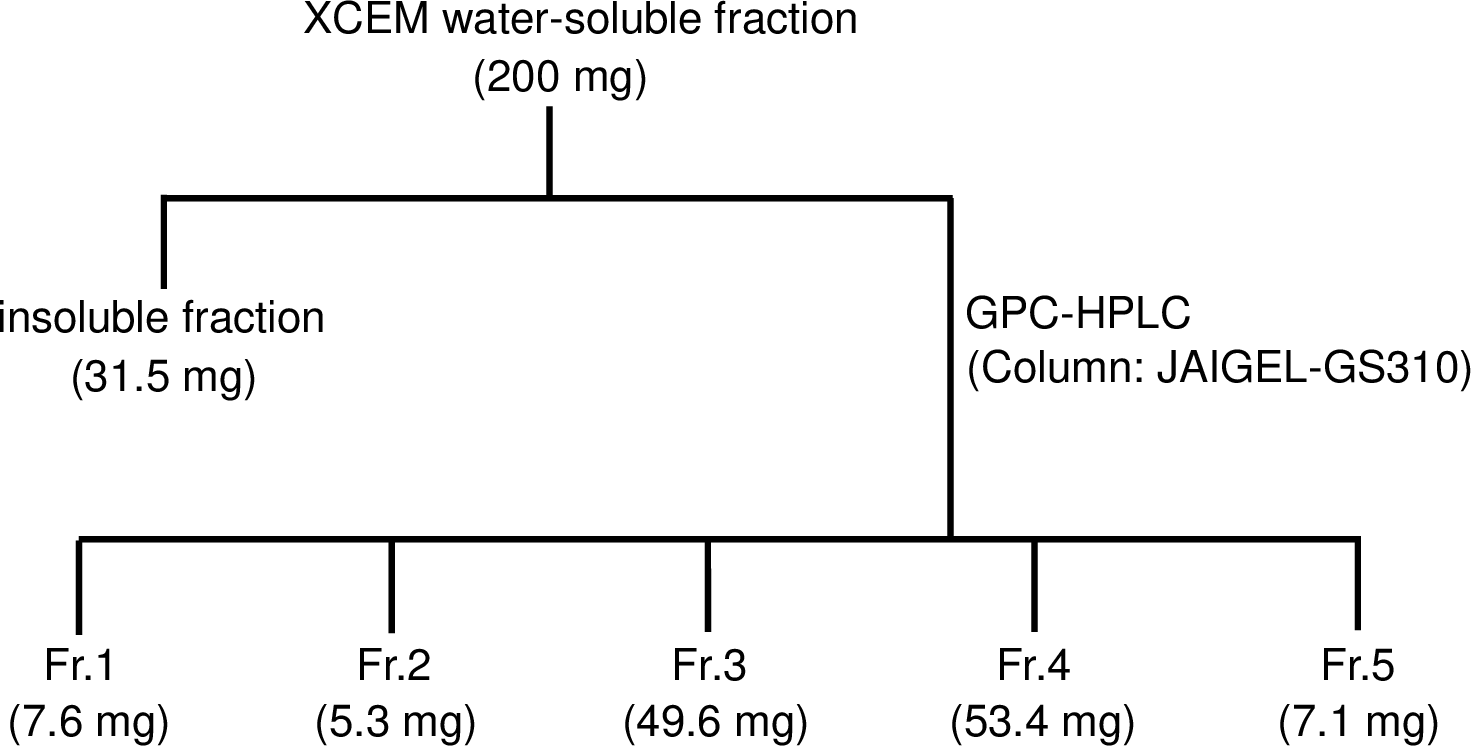

Supplement: S2 Fig — Two hundred g of dried water-soluble fraction were dissolved in 10 mL of water-methanol (1:4) mixture, and the solution was filtered through filter paper to give the filtrate and the insoluble (31.5 mg). The filtrate was subjected to preparative HPLC (column, JAIGEL-GS310 (20 mm x 500 mm, Japan Analytical Industry, Co. Ltd.). The column was eluted with a mixture of water-methanol (1:4) to give five fractions. (TIF) [file pone.0259297.s002.tif]

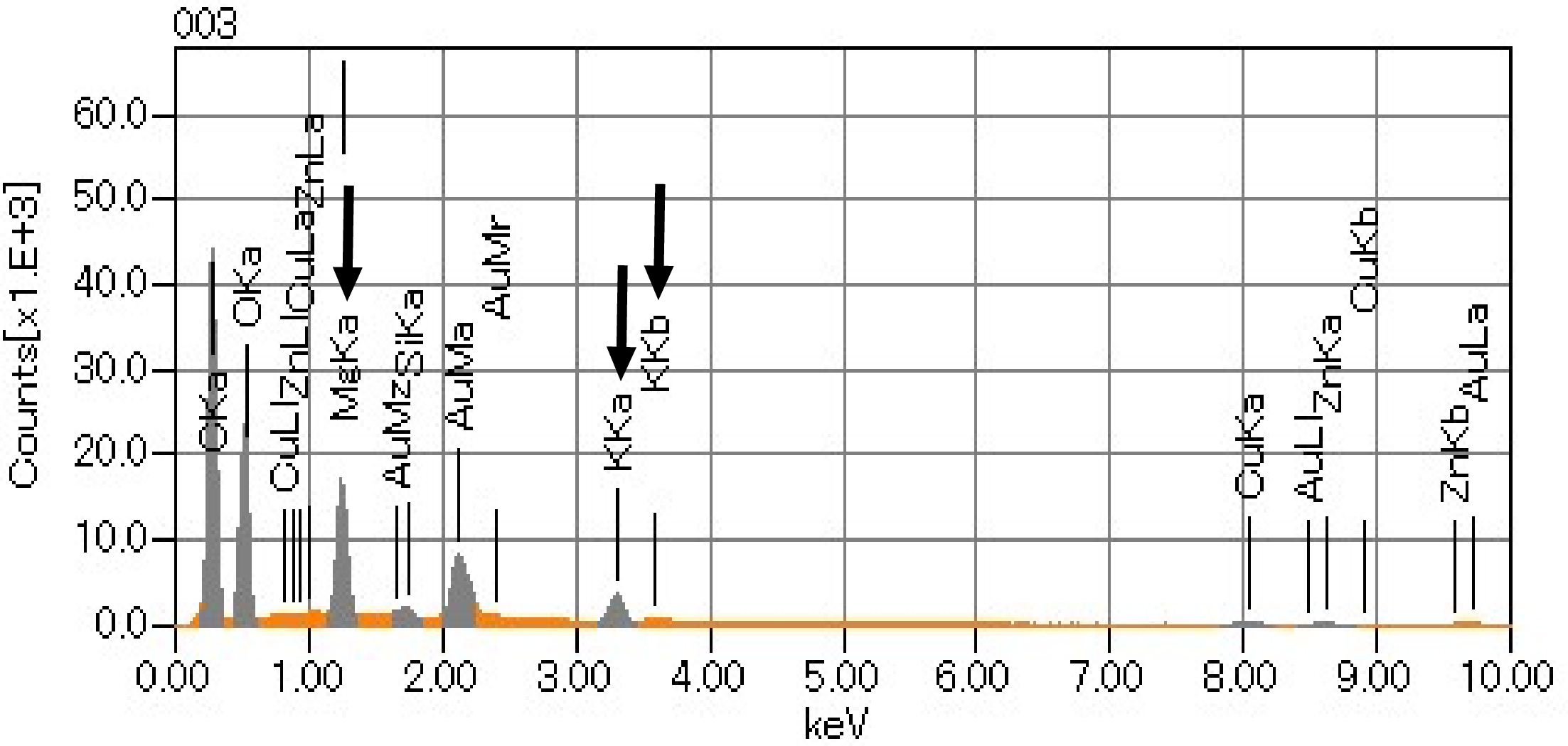

Supplement: S3 Fig — The downward arrows point to signals of Mg and K. Other signals are from the background. (TIF) [file pone.0259297.s003.tif]

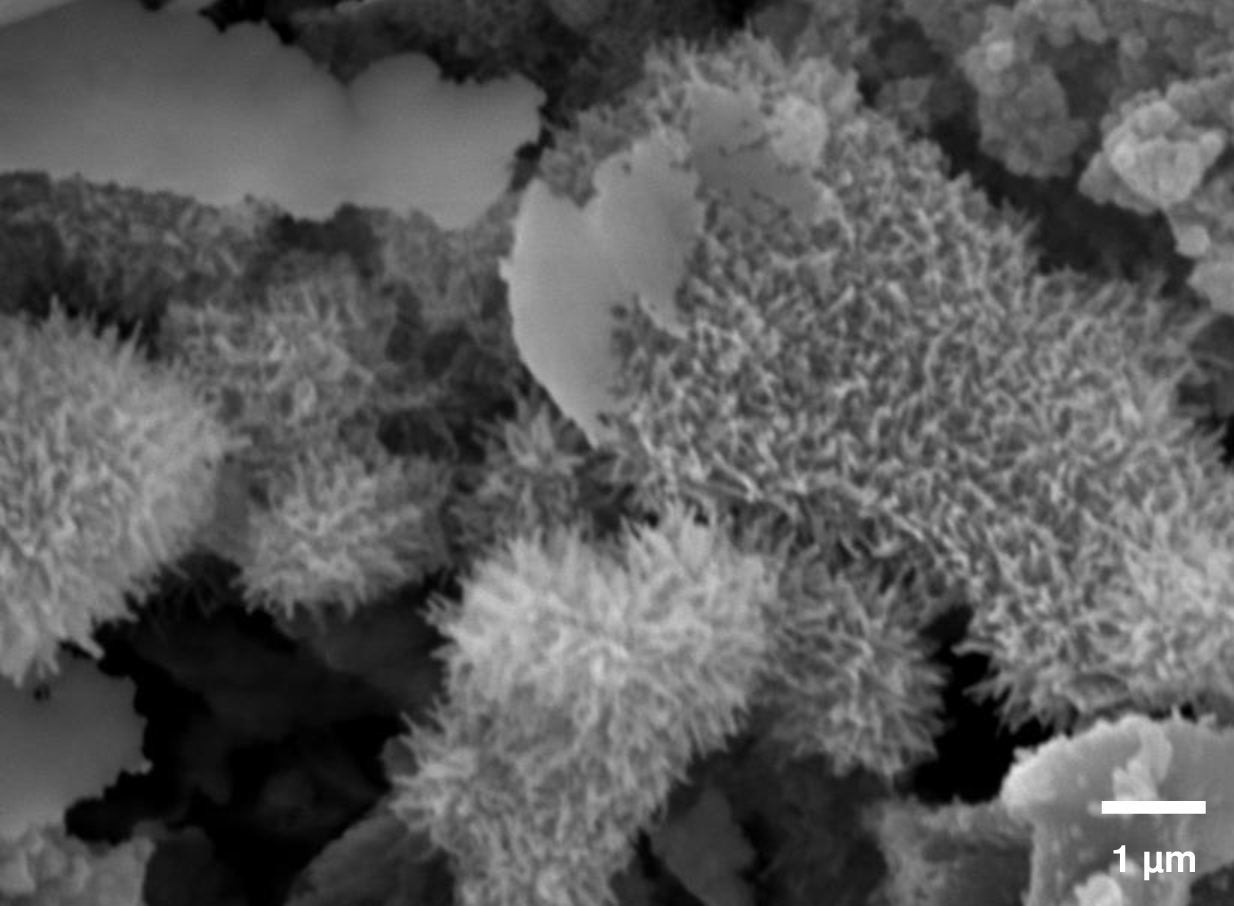

Supplement: S4 Fig — Note the exposed mesh-like structures. (TIF) [file pone.0259297.s004.tif]
