# Supplementary material for: Mg, K-containing microparticle: A possible active principle of a culture extract produced by a microbial consortium
Source: PLoS One. 2021 Nov 3;16(11):e0259297. doi: 10.1371/journal.pone.0259297 (PMC8565762; doi:10.1371/journal.pone.0259297)
Supplement: Attachment — Submitted filename: Response to Reviewers7.docx [file pone.0259297.s007.docx]

Academic Editor:

Comment: There are several point mentioned and addressed by the reviewers that need to be corrected in a revised version. I also recommend careful language editing before resubmission.

Reply: Yes, we did it.

Comment: For details on the required changes please carefully address all points raised be the reviewers. Because it may not be clear from the comment from reviewer one, I like to add that the authors should add and discusse more carefully the state of the art regarding possible mechanisms.

Reply: In the revised manuscript, we discussed to some extent the state of art regarding the possible mechanisms.

Journal Requirements:

Comment: Thank you for stating the following financial disclosure:

Reply: We stated the financial disclosure ‘This study was supported in the form of a grant awarded from the University of Tsukuba to HK.’

Comment: At this time, please address the following queries:

a) Please clarify the sources of funding (financial or material support) for your study. List the grants or organizations that supported your study, including funding received from your institution.

Reply: Our study was funded from a grant awarded from the University of Tsukuba to HK.

Comment: State what role the funders took in the study. If the funders had no role in your study, please state: “The funders had no role in study design, data collection and analysis, decision to publish, or preparation of the manuscript.”

Reply: The funders had no role in study design, data collection and analysis, decision to publish, or preparation of the manuscript.

Comment: If any authors received a salary from any of your funders, please state which authors and which funders.

Reply: We did not receive any salary from any funders.

Comment: If you did not receive any funding for this study, please state: “The authors received no specific funding for this work.”

Reply: We got funding from a grant awarded from the University of Tsukuba to HK.

Comment: Please include your amended statements within your cover letter; we will change the online submission form on your behalf.

Reply: We did so as you suggested.

Comment: We note that you have included the phrase “data not shown” in your manuscript. Unfortunately, this does not meet our data sharing requirements. PLOS does not permit references to inaccessible data. We require that authors provide all relevant data within the paper, Supporting Information files, or in an acceptable, public repository. Please add a citation to support this phrase or upload the data that corresponds with these findings to a stable repository (such as Figshare or Dryad) and provide and URLs, DOIs, or accession numbers that may be used to access these data. Or, if the data are not a core part of the research being presented in your study, we ask that you remove the phrase that refers to these data.

Reply: We removed the sentence which includes “data not shown”.

Comment: We note that Figure(s) 5 in your submission contain copyrighted images. All PLOS content is published under the Creative Commons Attribution License (CC BY 4.0), which means that the manuscript, images, and Supporting Information files will be freely available online, and any third party is permitted to access, download, copy, distribute, and use these materials in any way, even commercially, with proper attribution. For more information, see our copyright guidelines: http://journals.plos.org/plosone/s/licenses-and-copyright.

Reply: Fig.5 and Supplementary Fig. 4 in the submitted manuscript are the raw photographic data which we obtained directly from the scanning electron microscope. However, in order not to invite unnecessary confusion or misunderstanding, we removed the bottom end of the photograph and inserted a scale bar of 1 μm to Fig.5 and Supplementary Fig. 4.

Comment: Please include captions for your Supporting Information files at the end of your manuscript, and update any in-text citations to match accordingly. Please see our Supporting Information guidelines for more information: <http://journals.plos.org/plosone/s/supporting-information>.

Reply: Done as requested.

Reviewers' comments:

Reviewer's Responses to Questions

Reviewer #2:

Comment 1: They use the term EM for Effective Microorganisms. This abbreviation is not necessary since there are only 2 words. EM commonly refers to Electron Microscopy. It is rather confusion to read EM but it refers to something totally different. The legendary Francis Crick advised this reviewer 30 years ago to avoid use all unnecessary abbreviations and acronyms, so the readers can understand the articles better and avoid confusions.

Reply 1: EM is not simply an abbreviation. EM is a registered trademark and a brand name owned by EMRO (EM Research Organization). EM refers to a synthetic microbial consortium and is used over 100 countries. We already used this word, EM, in our previous paper (Ref.15 of this manuscript). On visiting web site emrojapan.com, more information about EM is available. Accordingly, we changed line 263 -265 as follows.

“EM represents a short form of Effective Microorganisms and is a registered trademark and a brand name owned by EMRO (EM Research Organization). EM consists mainly of photosynthetic bacteria, lactic acid bacteria and yeast.”

Comment 2: Please write out ECEM on line 83, page 5.

Reply 2: In compliance with the comment of Reviewer #2, we left out the term ECEM.

Comment 3: Please write out CMC (carbon microcoils) on line 238, page 9. CMC commonly refers to critical micelle concentration.

Reply 3: The term CMC for Carbon Micro Coil is an abbreviation made not by us, but was coined by its inventor, S. Motojima. Ref. 20 of this manuscript shows how it was termed. Accordingly, we changed line 238 (236 in the revised manuscript) as follows.

“carbon micro coil (CMC)[20].”

Comment 4: Please add more figure legend to Figure 5, provide more information about the microparticle, why the sizes cross a wide range. Do they all have similar elemental contents?

Reply 4: In compliance with the suggestion by Reviewer #2, we provided more information about the microparticle in the figure legend to Fig.5.

Comment 5: On lines 183-184, they wrote “The result was exactly as shown in Fig. 6”. Please replace the word exactly with “similar” since the results in Figure are not exactly, but similar. For example, in Panel A, E20 is ~33% and in Panel B, the E20 is ~38%. They are similar but not exactly identical.

Reply 5: In compliance with the suggestions by Reviewer #2, we replaced the word “exactly” with “similar” in the revised manuscript.

Comment 6: On line 221, page 9, they wrote “we have no idea regarding the functional role and the state of being of potassium……”. Please change to “in the current study, we did not focus on the study of potassium”.

Reply 6: In compliance with the suggestion by Reviewer #2, we changed the wording in the revised manuscript.

Question: PLOS authors have the option to publish the peer review history of their article (what does this mean?). If published, this will include your full peer review and any attached files.

Reply: No
