# Supplementary material for: Mg, K-containing microparticle: A possible active principle of a culture extract produced by a microbial consortium
Source: PLoS One. 2021 Nov 3;16(11):e0259297. doi: 10.1371/journal.pone.0259297 (PMC8565762; doi:10.1371/journal.pone.0259297)
Supplement: Attachment — Submitted filename: Response to Reviewers.docx [file pone.0259297.s008.docx]

October 15, 2021

*PLoS One*

Dear Dr. Stefan Wölfl:

Please find herewith our revised manuscript entitled “Mg, K-containing microparticle: a possible active principle of a culture extract produced by a microbial consortium” by Toru Higashinakagawa, Haruhisa Kikuchi and Hidekazu Kuwayama.

In compliance with the Editor’s suggestion, we stated in Introduction that EM is a registered trade mark and a brand name owned by EMRO (EM Research Organization). We stated that this work was initiated based on genuine scientific curiosity, since many papers on EM thus far published described only phenomenological observations. We were truly curious to know how EM works in a more simple system like cellular slime mold. This point was stated in the third paragraph of the introduction in the revised manuscript.

The Editor also asked about the intension of our work. To answer this question, I will describe how this work started and then proceeded. Almost 10 years ago, Kuwayama, the 3^rd^ author of the present paper, expressed his interest in EM•XGOLD which was/is freely available on the market. Kuwayama worked on cellular slime mold *Dictyostelium discoideum* for many years, and had been looking for any biological substances which may affect the life cycle of *Dictyostelium discoideum*. He had heard the rumor about EM•XGOLD and asked me to collaborate with him for looking at the effect of EM•XGOLD on the life cycle of this slime mold. I was then just retired from Waseda University, Tokyo, but still got a lab space at Tokyo Women’ Medical University. Thus, our joint work started in 2010. Since my place at TWMU did not have any facilities to grow *Dictyostelium discoideum*, most experiments were carried out in Kuwayama’s lab, 3 hours’ train ride away. In 2019, our first paper along this line was accepted in Journal of Molecular Microbiology and Biotechnology (JMMB, 29, 35-42, 2019). The JMMB paper described the differentiation promoting activity, namely promotion of fruiting body formation, in *Dictyostelium discoideum*, among other interesting effects. We found those observations quite interesting and proceeded to identify what the active principle of EM•XGOLD really is. At this point, we asked Kikuchi, the 2^nd^ author of the present manuscript, to join our group. Kikuchi was/is an organic chemist and contributed to the identification of biologically active substances from a variety of bio-organisms including *Dictyostelium discoideum*. Thus, our joint work started for identifying the active principle of EM•XGOLD which lead to the present manuscript.

As described above, our work is genuinely based on the scientific curiosity. We have never intended to advertise EM•XGOLD to the society. Moreover, our result refers only to cellular slime mold *Dictyostelium discoideum*, never meaning to address humans. Accordingly, we do not think the information inherent in this manuscript would not affect the market situation.

We hope the present manuscript will meet the publication in PLOS One.

Thank you for your consideration. I look forward to hearing from you.

Sincerely,

Dr. Toru Higashinakagawa

International Center for Molecular, Cellular and Immunological Research

Tokyo Women’s Medical University

8-1 Kawada-cho, Shinjuku

Tokyo 162-8666, Japan

TEL: +81-3-3353-8112 ( ext. 24062 )

FAX: +81-3-3352-3088

E-mail: toru@waseda.jp
