# Supplementary material for: Mg, K-containing microparticle: A possible active principle of a culture extract produced by a microbial consortium
Source: PLoS One. 2021 Nov 3;16(11):e0259297. doi: 10.1371/journal.pone.0259297 (PMC8565762; doi:10.1371/journal.pone.0259297)
Supplement: Attachment — Submitted filename: Referee report for PONE 6-2021.docx [file pone.0259297.s006.docx]

Referee report for PONE-D-21-17352

Higashinakagawa et al, *Mg, K-containing microparticle: a possible active principle of a culture extract produced by a microbial consortium*.

Higashinakagawa *et al* reported a very interesting and intriguing study and explain how simple elements have shown significant biological activity, namely, as they called the Effective Microorganisms (EM) on life cycle of *Dictyostelium discoideum*. They carried out systematic and careful studies of such EM･XGOLD, which is a well-known and popular health drink in Japan. This study is a continuation from their report in 2019 that “*The life cycle of Dictyostelium discoideum is accelerated via MAP kinase cascade by a culture extract produced by a synthetic microbial consortium*.”

They used various methods including i) Liquid-liquid partition, ii) column chromatography, iii) electron microscopy, iv) elemental analysis using Energy Dispersive X-ray (EDX) Spectroscopy, v) acid treatment and alkali pH titrations to figure out the major component of the biological activity. Through their careful observations and detailed studies, they conclude that the Mg, K-containing microparticle biological effect could be due to MgCO3. Although intriguing, their careful data and systematic analysis justify their conclusion. They may have solved one of long-standing puzzles in the popular health drink in Japan.

Some minor points:

1) They use the term EM for Effective Microorganisms. This abbreviation is not necessary since there are only 2 words. EM commonly refers to Electron Microscopy. It is rather confusion to read EM but it refers to something totally different. The legendary Francis Crick advised this reviewer 30 years ago to avoid use all unnecessary abbreviations and acronyms, so the readers can understand the articles better and avoid confusions.

2) Please write out ECEM on line 83, page 5.

3) Please write out CMC (carbon microcoils) on line 238, page 9. CMC commonly refers to critical micelle concentration.

4) Please add more figure legend to Figure 5, provide more information about the microparticle, why the sizes cross a wide range. Do they all have similar elemental contents?

5) On lines 183-184, they wrote “The result was exactly as shown in Fig. 6”. Please replace the word exactly with “similar” since the results in Figure are not exactly, but similar. For example, in Panel A, E20 is ~33% and in Panel B, the E20 is ~38%. They are similar but not exactly identical.

6) On line 221, page 9, they wrote “we have no idea regarding the functional role and the state of being of potassium……”. Please change to “in the current study, we did not focus on the study of potassium”.

After they make these corrections and changes, this reviewer highly recommends publication in PLoS ONE.
